# Medication Adherence Reminder System for Virtual Home Assistants: Mixed Methods Evaluation Study

**DOI:** 10.2196/27327

**Published:** 2021-07-13

**Authors:** Cynthia F Corbett, Elizabeth M Combs, Peyton S Chandarana, Isabel Stringfellow, Karen Worthy, Thien Nguyen, Pamela J Wright, Jason M O'Kane

**Affiliations:** 1 College of Nursing University of South Carolina Columbia, SC United States; 2 Center for Advancing Chronic Care Outcomes through Research and Innovation College of Nursing University of South Carolina Columbia, SC United States; 3 College of Engineering and Computing University of South Carolina Columbia, SC United States; 4 Arnold School of Public Health University of South Carolina Columbia, SC United States; 5 South Carolina Honors College Columbia, SC United States

**Keywords:** medication adherence, medication, virtual home assistants, virtual assistant, public health, health care costs, Echo device, device usability, digital health, mobile phone

## Abstract

**Background:**

Medication nonadherence is a global public health challenge that results in suboptimal health outcomes and increases health care costs. Forgetting to take medicines is one of the most common reasons for unintentional medication nonadherence. Research findings indicate that voice-activated virtual home assistants, such as Amazon Echo and Google Home devices, may be useful in promoting medication adherence.

**Objective:**

This study aims to create a medication adherence app (skill), MedBuddy, for Amazon Echo devices and measure the use, usability, and usefulness of this medication-taking reminder skill.

**Methods:**

A single-group, mixed methods, cohort feasibility study was conducted with women who took oral contraceptives (N=25). Participants were undergraduate students (age: mean 21.8 years, SD 6.2) at an urban university in the Southeast United States. Participants were given an Amazon Echo Dot with MedBuddy—a new medication reminder skill for Echo devices created by our team—attached to their study account, which they used for 60 days. Participants self-reported their baseline and poststudy medication adherence. MedBuddy use was objectively evaluated by tracking participants’ interactions with MedBuddy through Amazon Alexa. The usability and usefulness of MedBuddy were evaluated through a poststudy interview in which participants responded to both quantitative and qualitative questions.

**Results:**

Participants’ interactions with MedBuddy, as tracked through Amazon Alexa, only occurred on half of the study days (mean 50.97, SD 29.5). At study end, participants reported missing their medication less in the past 1 and 6 months compared with baseline (*χ^2^_1_*=0.9 and *χ^2^_1_*=0.4, respectively; McNemar test: *P*<.001 for both). However, there was no significant difference in participants’ reported adherence to consistently taking medication within the same 2-hour time frame every day in the past 1 or 6 months at the end of the study compared with baseline (*χ^2^_1_*=3.5 and *χ^2^_1_*=0.4, respectively; McNemar test: *P*=.63 and *P*=.07, respectively). Overall feedback about usability was positive, and participants provided constructive feedback about the skill’s features that could be improved. Participants’ evaluation of MedBuddy’s usefulness was overwhelmingly positive—most (15/23, 65%) said that they would continue using MedBuddy as a medication reminder if provided with the opportunity and that they would recommend it to others. MedBuddy features that participants enjoyed were an external prompt separate from their phone, the ability to hear the reminder prompt from a separate room, multiple reminders, and verbal responses to prompts.

**Conclusions:**

The findings of this feasibility study indicate that the MedBuddy medication reminder skill may be useful in promoting medication adherence. However, the skill could benefit from further usability enhancements.

## Introduction

### Background

Medication adherence, defined as taking medicines according to agreed-upon decisions between prescribing health care professionals and patients [1,2], is a major public health challenge. Research findings indicate that 50% or fewer people adhere to agreed-upon medication regimens [1,3]. Medication nonadherence contributes to suboptimal health management and increases overall health care costs among patients with chronic conditions [4,5]. The reasons for medication nonadherence are multifactorial [6] but often divided into intentional (eg, believing that medication is not needed or effective and stopping because of side effects) and unintentional (eg, inability to pay for medication, lack of understanding of how to properly take medications, and forgetfulness) [7]. Among unintentional reasons for medication nonadherence, forgetting to take medicine is one of the most common reasons [8-10]. Findings from a meta-analysis of medication adherence interventions among adults demonstrated that linking medication taking with existing daily routines and using behavioral strategies (eg, prompts to take medication) are the most effective strategies to promote adherence [11].

Technological advances, such as smartphones and electronic pillboxes, have been increasingly used as medication reminders [12-15]. For example, there are several smartphone apps that target disease management, including medication adherence, for people with diabetes, HIV, cancer, and other chronic conditions [16-20]. Improved rates of medication adherence have been demonstrated while using smartphone apps [16-19]. Key features of effective technological interventions include early participant input in the process to improve usability, a direct line between the patient and the people developing the technology along with the health care provider, a clear and easy-to-use interface, and the use of preexisting screening tools to measure medication adherence [20,21].

### Study Objective

Research findings indicate that voice-activated virtual home assistants (VHAs) such as Amazon Echo and Google Home devices may be useful in promoting medication adherence [22,23]. Amazon currently holds more than 70% of the market share of VHAs [24,25]. Software associated with Amazon Echo VHAs are commonly referred to as *skills*. Several medication adherence skills are available for use with Echo devices [26]. However, no study has specifically evaluated users’ perceptions of their effectiveness. Beaney et al [22] suggested that Amazon’s medication reminder skill could benefit from more development. For example, it does not have a feature for *as-needed* medications, that is, those not taken on a regular schedule [22]. Furthermore, Amazon’s general medication reminder skill provides only 1 reminder and does not allow the user to record whether the medicine was taken as prescribed. In this context, the primary purpose of this pilot project is to assess the usability and usefulness of a new medication-taking reminder skill, MedBuddy, developed by our team for use with Amazon Echo devices.

## Methods

### Study Design

A single-group mixed methods cohort study was conducted to evaluate the usability and usefulness of the MedBuddy medication reminder skill. Usability and usefulness are related properties of human-system interaction, which in combination determine human usage and satisfaction with the system [27], in this case, the MedBuddy skill for the Amazon Echo devices. Although a variety of properties have been associated with usability (eg, learnability, efficiency, and effectiveness), we defined it more broadly as participants’ likes and dislikes and whether they used the medication reminder system. Our definition of usefulness was pragmatic in investigating whether participants perceived the skill to improve medication adherence and whether they would continue its use and recommend others to use it.

### Skill Development and Design

**Figure 1:**
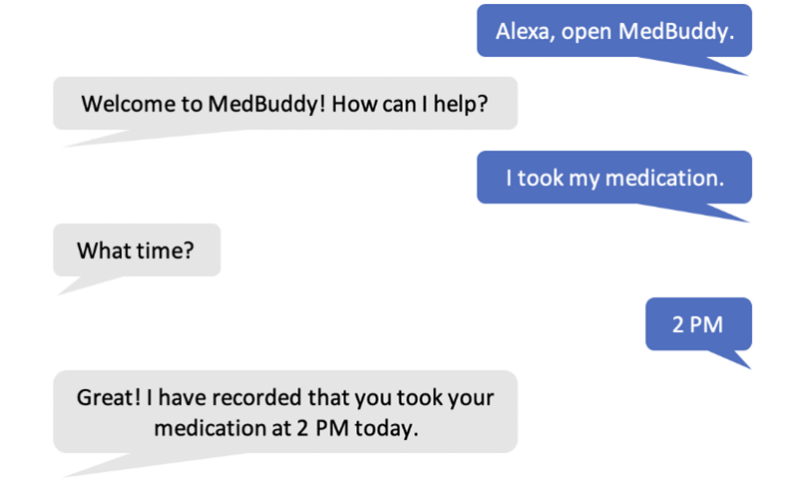
Example of participant dialogue with MedBuddy through Echo.

MedBuddy is a skill developed by our team for use with Amazon Echo devices. MedBuddy alerts users to take their medicines at a consistent time every day. During the development process, two aspects of usability and usefulness were considered: conversational experience with the skill and medication adherence documentation. The skill was designed for user interactions with the Echo device to be intuitive and modeled on the process of a natural conversation between 2 people. For the MedBuddy skill, the person using it starts the conversation, and then the Echo device, referred to as *Alexa*, responds (Figure 1). The skill tracks whether participants reported taking or missing their medicine and, if the medication was reported as taken, documents the time. Documentation about self-reported medication adherence and time was recorded when the participant activated the MedBuddy skill and said phrases such as “I took my medication” or “I forgot my medication.”

The skill was designed for each participant to receive three separate alerts to take their medication each day. Three alerts were chosen because, during alpha testing among our research team, more than 3 alerts were deemed bothersome, which could negatively affect the user experience. The first alert was set to take place 15 minutes before the participant’s desired medication-taking time. After the first alert, if the participant told MedBuddy through Echo that they took their medication, no subsequent alerts would occur. If the participant did not respond, a second alert occurred at the participant’s desired medication-taking time. If there was still no response from the participant, the third alert was provided 15 minutes later.

### Recruitment and Enrollment

Our team envisioned MedBuddy to be particularly useful for adults who spend most of their time at home. However, we conducted the initial usability and usefulness tests of MedBuddy with women who were college students and took oral contraceptives. We selected this population because (1) younger adults are likely to have more technological literacy than middle-aged or older adults and therefore may provide higher quality feedback about the skill with minimal frustration if it does not work as expected, (2) nonadherence to oral contraceptives is common [28,29], and (3) oral contraceptives are most effective when taken at approximately the same time every day [30]. Following the receipt of an exempt determination by the institutional review board, potential participants were recruited from an urban public university campus in the Southeastern United States. Recruitment flyers were distributed through electronic billboards, an electronic learning management system, and social media. In addition, an announcement about the research opportunity was presented to approximately 100 students who attended a university program orientation. Snowball sampling also occurred by enrolling referrals from current participants or our team’s social contacts. Potential participants were directed to contact the study coordinator by phone or email. When contacted, the study coordinator explained the study purpose, potential risks, and benefits and screened the participants for eligibility. Eligibility criteria included (1) currently taking an oral contraceptive but self-reporting difficulty remembering to take the medication some days or at about the same time each day, (2) living off-campus (because of internet inconsistency or internet overload in campus housing), and (3) owning a smartphone. Students meeting the eligibility requirements were invited to participate, and, for those interested, an enrollment appointment was scheduled.

In-person enrollment took place between February 1, 2020, and March 6, 2020, at a research center on a university campus. Volunteer participants provided verbal informed consent and then completed a baseline demographic and medication information questionnaire and a 4-item questionnaire about their adherence to their prescribed oral contraceptive medicine (Multimedia Appendix 1). Each participant received an Amazon Echo Dot device and a study account for the MedBuddy skill. Participants were then instructed on setting up and using the Amazon Echo Dot and subsequently the MedBuddy skill. Participants were first familiarized with activating the MedBuddy skill, using phrases such as “Alexa, open MedBuddy.” Once participants were familiar with activating the skill, the phrases for informing MedBuddy the medication had been taken or forgotten were demonstrated to participants. These phrases included “Alexa, tell MedBuddy I took my medication at 2:00 PM today” and “Alexa, tell MedBuddy I forgot my medication.” Participants were then given the opportunity to interact with Alexa and MedBuddy. Participants were also given the option of connecting the Alexa app on their smartphone to the study account, which allowed them to interact with MedBuddy through either the Echo Dot or their smartphone. Participants received a US $15 honorarium at the completion of the baseline visit.

### Measures

Participants’ demographic and medication-related information were collected using a baseline survey. Participants’ responses to the prompts provided by MedBuddy were tracked by the research team. We tracked whether participants reported taking or missing their medicine or whether they failed to respond to the reminder. Participants also self-reported their perceived adherence to their oral contraceptives more globally by answering four questions at baseline and at the end of this study (Multimedia Appendix 1). The adherence perception questions were developed by the authors and not psychometrically tested before their use in this feasibility study. Additional usability and usefulness data were collected during structured exit interviews conducted by telephone after each participant used MedBuddy for at least 60 days (Figure 1). Participants’ responses to the telephone interview questions were documented on an interview form. Notes taken by the research team in response to the open-ended questions were read back to each participant to confirm that the team member’s documentation of their responses was accurate. Participants were sent a US $25 honorarium following the completion of the poststudy interview.

### Analyses

Participants’ demographic characteristics and medication information about their oral contraceptives were summarized using descriptive statistics. Participants’ verbal responses to the Echo device, as they used the MedBuddy skill, were summarized using frequencies based on 60 days of use. We also evaluated participants’ responses to MedBuddy throughout the study time. Specifically, a paired two-tailed *t* test was conducted to evaluate whether there was a significant reduction in participants’ responses to MedBuddy during the first 2 weeks of the study versus the last 2 weeks of the study. Participants’ self-reported medication adherence between baseline and the study’s conclusion was evaluated using the McNemar chi-square test. All statistical analyses were completed using the SPSS (version 27; IBM Corp) with an *α* value set at .05. Data from the open-ended structured interview questions were qualitatively categorized into evaluative topics to summarize participants’ perceptions of the usability and usefulness of MedBuddy.

## Results

### Demographic and Medicine-Taking Characteristics

Among the participants, 92% (23/25) completed the full study. The COVID-19 pandemic necessitated campus closure during the study, which resulted in an inability to follow up with 2 participants. We did not detect differences in demographic characteristics between those who did and did not complete the study. The sample was characterized by heterosexual, White women who had a mean age of 21.8 (SD 6.2) years, and none were married, but all lived with one or more other people. Most participants (14/25, 56%) started their medication week on Sunday, which is important concerning adherence because most oral contraceptives are taken for 3 consecutive weeks followed by 1 week of a placebo each month. The participants’ desired time to take the medication varied, but most women took their medicine at night before bed or in the morning. Interestingly, all the women identified at baseline that they currently used a medication reminder method, with most (18/25, 72%) stating that they used a smartphone in conjunction with a reminder app or setting a smartphone alarm. Thus, MedBuddy was an adjunct or replacement of the reminder strategy currently used. Participants’ demographic and medication-related characteristics are summarized in Table 1.

**Table 1:** Participants’ demographic and medicine-taking characteristics (N=25).

Baseline demographics			Participants, n (%)
**Race**			
	White	20 (80)	
	Black	1 (4)	
	Asian	2 (8)	
	Mixed race	2 (8)	
**Sexual orientation**			
	Heterosexual	23 (92)	
	Bisexual	2 (8)	
	Homosexual	0 (0)	
**Medication time**			
	Morning	9 (36)	
	Afternoon	1 (4)	
	Evening	5 (20)	
	Night	10 (40)	
**Starting day**			
	Sunday	14 (56)	
	Monday	3 (12)	
	Tuesday	1 (4)	
	Wednesday	5 (20)	
	Friday	1 (4)	
	It changes	1 (4)	
**Current methods of medication adherence**			
	Significant other	3 (12)	
	Parents or family	1 (4)	
	Medication app	2 (8)	
	Calendar or planner	2 (8)	
	Phone alarms or reminders	17 (68)	

### MedBuddy Use

During the study, we tracked the participants’ verbal responses to MedBuddy prompts. Participants responded to MedBuddy prompts just a little more than half of the time (study days: mean 50.97, SD 29.5). The response rate ranged from 6.6% to 86.7%. MedBuddy use slightly declined from the first 2 weeks of the study (mean response 70.5%, SD 0.23) compared with the last 2 weeks of the study (mean response 59.2%, SD 0.27), (*t_23_*=2.4; *P*=.03). Only 1 participant responded that she missed medication. All other responses noted that the medication had been administered. There were no discernable differences in participant characteristics for those who responded to MedBuddy more regularly than those who had limited responses.

### MedBuddy Usability

After participants used MedBuddy for at least 60 days, we interviewed them to obtain information about their user experience. Most participants rated their overall experience with the Echo device as *good* or *very good* (18/23, 78%) and the remainder rated the experience as *neutral* (5/23, 22%)*.* Participants rated their overall user experience with the MedBuddy skill less positively, with 56% (13/23) rating it as *good* or *very good*, 35% (8/23) rating it as *neutral*, and 9% (2/23) rating it as *poor.* However, nearly all participants (21/23, 91%) rated MedBuddy as *effective* (7/23, 30%) or *very effective* (14/23, 61%) as a medication reminder, and the remainder indicated it was mostly ineffective (1/23, 4%) or ineffective (1/23, 4%). Most participants (15/23, 65%) said they would continue to use MedBuddy as a medication reminder if provided with the opportunity. The primary reasons participants wanted to continue using MedBuddy were that (1) the external alert separated from their phone was beneficial, (2) they could hear the alert if they were in a room different from the room where the Echo Dot was located, (3) the verbal prompts from a human-sounding voice were motivating, and (4) more than 1 reminder helped prompt them to take their medicine. For instance, a participant noted:

I really liked that I got three reminders because I almost always ignored the first two, but it was enough reminders to force me to do it.

Participant 110

Two other open-ended interview questions focused on the features and aspects of MedBuddy that participants liked or disliked. The features that participants liked aligned with the reasons why they would continue to use and recommend it: (1) receipt of multiple reminders 15 minutes apart, (2) the interface between their smartphone and the Echo Dot, and (3) verbal reminders with voice interaction. A typical participant response was “I like the verbal reminders, the vocal response and prompt” (Participant 112). Another participant stated:

My favorite was the verbal aspects, someone that I could hear, multiple reminders, the verbal aspect of responding was attractive.

Participant 107

The ability to verbally report the medication action instead of the need to locate another technological device (such as a smartphone) was perceived as convenient and beneficial.

The MedBuddy features that participants disliked were consistently noted by many. These features included (1) speech recognition difficulty such that the Echo Dot or smartphone Alexa app did not open the MedBuddy skill or required several commands to open it; (2) the need to alert the Echo Dot or smartphone multiple times to stop future prompts, again because of speech recognition difficulties; (3) logging errors (eg, if the medication was taken late in the evening, MedBuddy may not have recorded the response on an intended day, but for the next day, canceling alerts for the next day); and (4) minimal ability for the user interface. Regarding the latter, some participants reported a desire to view and edit their medication history through their smartphones.

### MedBuddy Usefulness

A large majority of participants (21/23, 91%) said they would recommend MedBuddy to someone who had difficulty with medication adherence. Participants stated that they would recommend MedBuddy to others because (1) it personally facilitated their own adherence; (2) helped them incorporate medication taking into their daily routine; and (3) the multiple reminders would benefit other people with trouble regarding medication adherence, including those who were older and may be at home more or those who take multiple medications. For example, a participant stated that she would have liked to have her grandmother use MedBuddy on an Echo device, stating:

I would recommend the MedBuddy skill because I live with some older people and they take multiple daily medications, and it would help them because it helped me.

Participant 109

Participants also answered questions about their perceived medication adherence before and after using the MedBuddy skill (Textbox 1). The 4-item adherence questionnaire had a Cronbach *α* of .77 at baseline (n=25) and .70 at the end of the study (n=23). Participants reported missing their medication less in the past 1 and 6 months at the end of the study compared with baseline (*χ^2^_1_*=0.9 and *χ^2^_1_*=0.4, respectively; McNemar test: *P*<.001 for both). However, there was no significant difference in participants’ reported adherence to consistently taking medication within the same 2-hour time frame every day during the past 1 or 6 months at the end of the study compared with baseline (*χ^2^_1_*=3.5 and *χ^2^_1_*=0.4, respectively; McNemar test: *P*=.63 and *P*=.07, respectively).

**Perceptions of medication adherence questions.**

**Questions**

1. In the past month, how often have you missed taking your birth control pill (1=never, 2=once or twice, 3=a few times, 4=several times, 5=frequently)? Please circle the appropriate response:
2. In the past month, how often would you say you have taken your birth control pill within the same 2-hour framework (1=almost never, 2=infrequently, 3=sometimes, 4=usually, 5=always)? Please circle the appropriate response
3. In the past 6 months, how often have you missed taking your birth control pill (1=never, 2=once or twice, 3=a few times, 4=several times, 5=frequently)? Please circle the appropriate response:
4. In the past 6 months, how often would you say you have taken your birth control pill within the same 2-hour framework (1=almost never, 2=infrequently, 3=sometimes, 4=usually, 5=always)? Please circle the appropriate response:

## Discussion

### Study Context

The purpose of this study was to evaluate the use, usability, and usefulness of MedBuddy, a medication adherence skill designed for Amazon Echo devices. Participants’ recorded responses indicated that their use of MedBuddy varied widely. Participants were enrolled in February and early March 2020; many of them left their residences to travel for a spring break. Unexpectedly, the university transitioned to web-based learning directly after spring break. As a result, many of the participants were separated from their Echo devices and could not use MedBuddy. Most participants subsequently returned to their residences or downloaded the Alexa app on their phones and could resume using MedBuddy. While MedBuddy was used more frequently in the last 2 weeks of the study compared with the last 2 weeks of the study, we do not attribute the lack of use to disinterest or dissatisfaction with the skill, especially given the participants’ positive perspectives on usability and usefulness during the poststudy interviews.

### Usability Findings

Most participants reported at least a *good* user experience with the skill. The primary dissatisfaction with the skill was when the participant asked Echo to either open the skill or tell (the skill) their medicine was taken (or missed), Echo had difficulty interacting with MedBuddy because of speech recognition. For instance, when some participants instructed Echo to *open MedBuddy*, Echo would open a different skill with the word *buddy* in the title. Similarly, a voice response indicating the medicine was taken or missed, which should cancel future reminders for the day, was sometimes not recorded because Echo did not recognize and communicate with the right skill. In these instances, participants received one or two additional reminders from MedBuddy after having said they had taken their medication. A participant noted that she continued to use MedBuddy because it helped her remember to take her medicine, but she stopped even trying to communicate with MedBuddy through Echo because of these described speech recognition interface problems.

Despite the speech recognition errors with the skill, participants requested more ways to interface with MedBuddy both before and after the university enacted remote learning. Participants could communicate through MedBuddy on their phone if they activated the Alexa app on their phones, which some did when they enrolled in the study and others did later during the study. Consequently, participants could communicate with Echo and MedBuddy, even if they were not currently at home. Some participants requested to see their medication history, a feature that was not available. However, our team was presumptively considering adding this feature to the second-generation version of the skill.

### Usefulness Findings

Nearly all participants perceived MedBuddy as useful for medication adherence, which was particularly encouraging because all participants reported using some other reminder at baseline, with most using mobile phones as reminders. MedBuddy was reported to be useful despite the existing reminder system. A MedBuddy feature frequently mentioned as helpful was the automatic receipt of three reminders, which made the reminder more difficult to ignore than, for instance, receiving one reminder or an alarm via their mobile phones. In addition, several participants stated that reminders sounding like a human voice made them more responsive and accountable to the system. This finding resonates with other research showing that some users personify voice-activated devices [23,31]. Furthermore, being able to respond to MedBuddy using a voice command appealed to some users. The participants found the hands-free use efficient, which has also been reported among people who use VHAs for other purposes [32,33]. More than 90% (21/23, 91%) of participants reported that MedBuddy helped them take their medicine as prescribed. In the self-reports of medication adherence, participants reported missing their medicine less in the last 1 and 6 months in the poststudy versus prestudy assessment. However, there was no baseline versus poststudy difference in the frequency by which they took their medicine within the desired 2-hour time frame. More than 65% (15/23) of participants stated that they would continue using MedBuddy if given the opportunity. Several of those who declined to continue MedBuddy use stated that MedBuddy helped them develop habits for a better medicine-taking routine, so they had no need for future use.

Finally, more than 90% (21/23, 91%) of participants stated that they would recommend MedBuddy to others because they thought it would help with medication adherence as it had helped them. Many participants thought MedBuddy would be ideal for older adults, people who require multiple medicines to manage health conditions, and those who spent most of the day at home. As participants were all students at the time of the study, they considered their schedules to be more hectic and less predictable than those who may be retired or at home when medications are due. These findings were especially salient because we envision adults and people who take multiple medicines as the primary target population of MedBuddy users.

### Study Limitations

This feasibility study had several limitations. We tested it with a small convenience sample consisting entirely of women, primarily young adults, White, and non-Hispanic college students living off-campus. Except for being women, participants’ demographic characteristics closely represented the larger university population. For example, the university population was 77% (24,049/31,232) White, whereas our sample was 80% (20/25) White. Furthermore, because of inconsistent internet service in campus housing, our participants all lived off-campus. Freshmen at the university are required to live on campus, but among nonfreshmen, 90% (22,484/24,982) live off-campus. We encountered an unexpected challenge in that some participants were separated from their Echo device because the university transitioned to all web-based classes in March 2020 because of the COVID-19 pandemic. The unexpected change highlighted the significance of the option to use the Alexa smartphone app as a communication tool between Echo and MedBuddy. Nevertheless, the findings about participants' MedBuddy use and its' use over time may not reflect use under normal circumstances. In addition, this study only tracked users for 60 days. Therefore, it is unknown whether people would continue to use and find the medication reminder system useful for a longer period. Self-reported measures for medication adherence were developed by the authors and did not have established psychometric properties. Furthermore, subjective measures of medication adherence are known to overestimate adherence [34]. Thus, the improved medication adherence found in this study must be interpreted cautiously.

### Future Directions and Implications

On the basis of the study usability findings, several improvements to MedBuddy have been made or are in progress. The skill’s name was changed to improve the speech recognition interface with Echo devices. We learned that there are several existing skills with the terms *med* or *buddy* as part of the skill name. Other skills’ similar names sometimes made it difficult for the Echo device to differentiate commands for *MedBuddy* versus a command for a different skill. As it seemed to be a problem for some participants and not problematic at all for others, we believe communication success may have varied based on voice tones and diction—an issue noted by users of other voice-activated devices [35]. Thus, we deemed it necessary to change the skill’s name. On the basis of naming tips for skills published on the web by Amazon, named skills and other software promoting medication adherence, and our interface tests between Echo and the skill, by using a short-list of potential names, MedBuddy was renamed as *Pill Minder*. Initial alpha tests have been positive with the new name, although excessive background noise may still interfere with device communication. To further evaluate the usefulness and accuracy of participants’ communication with the renamed skill, Pill Minder, we plan to use pill bottles that electronically record cap removal. We will analyze the relationship between participants’ responses to Pill Minder and pill bottle opening.

As recommended by participants, we are in the process of creating an interface that allows participants to view their personal medication history. In addition, users of our renamed skill, Pill Minder, will be able to authorize others, such as a support person, caregiver, or health professional, to see their medication use history. This change also aligns with the recommendations of participants from another study completed by our team involving dyads of older adults and their support persons [36], in which support persons indicated a desire for an Echo medication skill that enabled them to see whether their older adults had reported taking their medication. For research purposes, we will be able to view individual participants’ data or aggregated data in a more user-friendly format than was the case with the original MedBuddy user data. Finally, the new version of the skill, Pill Minder, has a more streamlined user-setup process.

Another major advancement in Pill Minder is the capacity for users to receive medication reminders multiple times a day. Users can select reminder times for as many different dosing times as needed, whereas the original MedBuddy only had the capacity for 1 reminder per day. These enhancements are consistent with recommendations in the literature suggesting that medication reminder systems for Echo could benefit from further development [22].

### Conclusions

Medication adherence continues to be a global public health challenge. It is a complex phenomenon, but research findings suggest that forgetting to take medicine is a factor that negatively impacts adherence for many people [8-10], including those who participated in this study. MedBuddy was designed as a medication reminder system that interfaces with Amazon Echo. The initial feasibility test was positive. Participant feedback on the MedBuddy skill usefulness was particularly positive. Overall, the most notable features were multiple verbal reminders, with the capacity for a verbal response, and receipt of reminders external to the mobile phone, but compatible with it through the Alexa app. Although most participants also positively evaluated usability, several suggestions were made to improve the skill. The next version of MedBuddy—renamed Pill Minder—has been designed with features to address study participants’ suggestions. Alpha testing has been initiated within our team, and further testing will be launched outside the team soon. In summary, the medication reminder skill developed by our team was perceived as useful but requires more refinement for better usability. Once refined, we will study the usefulness and usability of Pill Minder with adults who take medicine at least twice a day.

## Appendix

Multimedia Appendix 1 Poststudy usability and usefulness interview questions.

## Abbreviations

VHA: virtual home assistant
