# Correction: Medication Adherence Reminder System for Virtual Home Assistants: Mixed Methods Evaluation Study

**DOI:** 10.2196/36381

**Published:** 2022-01-27

**Authors:** Cynthia F Corbett, Elizabeth M Combs, Peyton S Chandarana, Isabel Stringfellow, Karen Worthy, Thien Nguyen, Pamela J Wright, Jason M O'Kane

**Affiliations:** 1 College of Nursing University of South Carolina Columbia, SC United States; 2 Center for Advancing Chronic Care Outcomes through Research and Innovation College of Nursing University of South Carolina Columbia, SC United States; 3 College of Engineering and Computing University of South Carolina Columbia, SC United States; 4 Arnold School of Public Health University of South Carolina Columbia, SC United States; 5 South Carolina Honors College Columbia, SC United States

In “Medication Adherence Reminder System for Virtual Home Assistants: Mixed Methods Evaluation Study” (JMIR Form Res 2021;5(7):e27327), three errors were noted.

Due to a system error, the name of one author, Cynthia F Corbett, was replaced with the name of another author on the paper, Elizabeth M Combs. In the originally published paper, the order of authors was listed as follows:

Elizabeth M Combs, Elizabeth M Combs, Peyton S Chandarana, Isabel Stringfellow, Karen Worthy, Thien Nguyen, Pamela J Wright, Jason M O'Kane

This has been corrected to:

Cynthia F Corbett, Elizabeth M Combs, Peyton S Chandarana, Isabel Stringfellow, Karen Worthy, Thien Nguyen, Pamela J Wright, Jason M O'Kane

In the originally published paper, the ORCID of author Cynthia F Corbett was incorrectly published as follows:

0000-0002-2254-6958

This has been corrected to:

0000-0003-2706-2116

In the originally published paper, the email of the Corresponding Author was incorrectly published as follows:

combsel@email.sc.edu

This has been corrected to:

corbett@sc.edu

The correction will appear in the online version of the paper on the JMIR Publications website on January 27, 2022, together with the publication of this correction notice. Because this was made after submission to PubMed, PubMed Central, and other full-text repositories, the corrected article has also been resubmitted to those repositories.

