# Supplementary material for: Medication Adherence Reminder System for Virtual Home Assistants: Mixed Methods Evaluation Study
Source: JMIR Form Res. 2021 Jul 13;5(7):e27327. doi: 10.2196/27327 (PMC8317037; doi:10.2196/27327)
Supplement: Multimedia Appendix 1 [file formative_v5i7e27327_app1.docx]

Appendix 1

**Post-Study Usability and Usefulness Interview Questions**

1. How would you rate your overall experience with Alexa? (1 meaning very poor, 2 meaning poor, 3 meaning neutral, 4 meaning good, 5 meaning very good). Please circle the appropriate response:

1 2 3 4 5

1. How would you rate your overall experience with MedBuddy Skills? (1 meaning very poor, 2 meaning poor, 3 meaning neutral, 4 meaning good, 5 meaning very good). Please circle the appropriate response:

1 2 3 4 5

3. How many times did you miss your medication over the past 8 weeks? _______

4. What percentage of time did you get your reminders on the Echo Dot? _______

5. What percentage of time did you get your reminders on your smart phone? _______

6. How effective would you say MedBuddy was in aiding you in medication adherence?

_____not effective _____mostly ineffective _____ mostly effective _____effective

7. Do you plan to continue using MedBuddy in the future for medication adherence?

_____Yes _____No _____Unsure

Please provide rationale for your answer above:_______________________________________

8. Would you recommend MedBuddy to anyone with medication adherence issues?

_____Yes _____No _____Unsure

Please provide rationale for your answer above:_______________________________________

9. Describe your favorite aspects of using MedBuddy: ___________________________________

10. Describe aspects of MedBuddy that could be improved upon:___________________________
